# Modification of Biotesting-Based Fermented Dairy Product Design for Curd and Curd Products

**DOI:** 10.3390/foods11203166

**Published:** 2022-10-11

**Authors:** Zinaida S. Zobkova, Elena A. Yurova, Vladislav K. Semipyatniy, Ekaterina G. Lazareva, Daria V. Zenina, Irina R. Shelaginova

**Affiliations:** Russian Research Institute of the Dairy Industry, Lusinovskaya Str. 35, 115093 Moscow, Russia

**Keywords:** biotesting, *Tetrahymena pyriformis*, curd, cottage cheese, curd products, fermented dairy products, relative biological value

## Abstract

The key trends driving the global dairy market are shelf-life extension and generating consumer demand for new products. Healthy diets and special foods meet the criteria based on the protein digestibility-corrected amino acid score, while other factors affecting the digestibility and actual biological value of the protein are not considered. Express biological evaluation tests are very important for choosing the optimal formulation and efficient manufacturing process in order to maximize the biological value (BV). Such tests adequately represent the food properties: safety, nutrition value, digestibility, health benefits, etc. This study deals with the procedures for the quick biological evaluation of dairy products using indicator organisms. We adapted the relative biological value evaluation procedure, involving *Tetrahymena pyriformis*, for curd (cottage cheese) and curd products. The experiments showed that the most significant parameters are the milk pasteurization temperature and the curd heating temperature. The full factorial experiment identified the optimal curd production conditions to maximize the relative biological value (RBV): 81 °C milk pasteurization temperature and 54 °C curd heating temperature using the acid method of curd production. With these parameters, the RBV is at least 282%. Biotesting confirmed the optimal curd product component ratio of 60% curd to 40% fermented dairy beverage.

## 1. Introduction

The food market is changing; new products are being introduced, and conventional technologies are being upgraded [1,2,3]. The key food industry trends are shelf-life extension and generating consumer demand for new products. Therefore, new food quality criteria are required [4,5,6]. Any food product, regardless of its shelf life, should be safe and have a high biological value.

The global, commonly accepted biological value and food safety procedure is the study of higher animal metabolism in a laboratory [7]. This approach has some disadvantages; biotesting is long, and the cost is enormous.

Among other approaches to comprehensive food quality assessment with living organisms, one alternative is the study of protozoan metabolism and using protozoa as a test model for food biotesting [8,9,10,11,12,13,14]. An example of such an organism is *Tetrahymena pyriformis*. One of the most successful and promising models for establishing the nutritional value, harmlessness, and biological value of products is the *Tetrahymena pyriformis* infusoria. Despite its microscopic size (20–50 µm), this single-celled organism is very useful as a test object for a range of biological experiments. The weight of one cell is 1.5 ×$\;10^{-9}$ g. It contains (by dry weight) 6.2% total nitrogen (20 bound and 17 free amino acids), 4.5–4.9% nucleic acids, 15–20% glycogen, and 15–20% lipids. *Tetrahymena pyriformis* has a mouth, pharynx, digestive vacuoles, lysosomes, microsomal apparatus, and many enzyme and hormonal systems. It utilizes nitrogen in a more complex form than amino acids, which sharply distinguishes it from microbes. In the process of nutrition, *Tetrahymena pyriformis* undergoes acidic and alkaline stages of exposure to inclusions processed in digestive vacuoles. Enzymes are delivered by lysosomes. The main nitrogen-containing substances excreted by *Tetrahymena pyriformis* are: ammonia, glycine, glutamic acid, alanine, aspartic acid, and tyrosine. *Tetrahymena pyriformis* needs ten essential amino acids. It is able to consume intact proteins and synthesize certain fatty acids, as well as dispose of them from food. It also has hydrolytic activity related to a number of sugars. The duration of the general life cycle of *Tetrahymena* pyriformis is 4–6 h [15,16,17,18,19].

With regard to the use of *T. pyriformis* as a test object in the world, it is mainly used to assess the toxicity of various objects. Thus, the authors [20] propose a new type of bacterial toxicity assessment. This simple and quick test is able to detect bacteria producing toxic substances which may pose a biological hazard. It can also be used to assess the risk of microbes intended for targeted release. In addition, the toxicity of iron oxide and arsenic nanoparticles can be assessed using tetrachymenes [21]. The accumulation of As(V) in *T. pyriformis* leads to an increase in the content of trivalent arsenic as a result of saturation of the cellular ability to methylate arsenic and/or redox reactions. This exposure also leads to an imbalance between oxidants and antioxidants. This, in turn, leads to oxidative damage and cell death. Hajdu et al. [22] assessed soil toxicity using *Tetrahymena pyriformis*. The authors found that, in the complex system of soil and soil suspension used in bioanalysis, in addition to *Tetrahymena pyriformis*, the use of other biotesting approaches is effective. An interesting application of *Tetrahymena pyriformis* was proposed by R. Chaudhry and A. R. Shakoori [23]. In their study, the authors evaluated the potential of *Tetrahymena pyriformis* as a bioremediant of copper-saturated waters.

With regard to the use of infusoria for food testing, Dolgov et al. [11] developed guidelines for the express testing of feed and livestock biological value. Their study estimated *Tetrahymena pyriformis* vital activity metrics for subsequent analysis: survival rate, growth response, lag phase duration, population growth curve, cell size, grown biomass size, etc. Bogdan et al. [24] proposed approaches to food safety and biological value assessment with *Tetrahymena*. As part of the study, they ranked the food products by their physical and chemical compositions and energy value. Furthermore, the results were adjusted to the cultivation of *Tetrahymena pyriformis* to match human daily calorie intake. Bondaruk et al. [12, 25] tested two growth media used to study food’s biological value and safety with *Tetrahymena*. Zhurikhina et al. [26] studied the biological value of a serum protein enzymatic hydrolysate sample using *Tetrahymena pyriformis* test objects. They recommended using enzymatic hydrolysate for sports nutrition and as a component of healthy foods.

*Tetrahymena pyriformis* was used to examine the safety and relative biological value (RBV) of herbal beverages (Manchurian walnut) [27]. The studies showed that the protozoa generation and their cell sizes increased with storage time, but they did not significantly differ from the reference sample. Shulgin et al. [28] used infusoria to find that the RBV of aquatic life muscle tissue isolated proteins is significantly higher than that of non-isolated proteins. The lowest muscle tissue RBVs are caused by the anti-nutritional effect of proteins and non-protein components in aquatic organisms.

Zobkova et al. [29] used a biological model of *Tetrahymena pyriformis* to study the safety of transglutaminase-containing curd. The toxicity criterion was a decrease in the infusoria growth relative to the reference growth (%).

The cell count increase was estimated as the difference between the arithmetic mean cell numbers at the beginning and end of the experiment.

The toxicity criterion *E* (%) was estimated as:

(1) $$E=\frac{N_{tp}}{N_{c}}\cdot 100,$$

where $N_{tp}$ is the infusoria count in the tested product at the end of the experiment, pcs; and $N_{c}$ is the infusoria count increase in the reference sample at the end of the experiment, pcs.

The toxicity categories determined from the *Tetrahymena* culture changes were as follows:

- non-toxic: more than 100%;
- weakly toxic: 50–100%;
- toxic: less than 50%.

The study concluded that the fermented transglutaminase-containing curd sample was not toxic.

The authors of paper [29] studied several factors affecting yogurt RBV and developed guidelines for creating fermented dairy products with an optimal biological value [30]. They also developed express RBV tests for fermented dairy products using *Tetrahymena pyriformis* test organisms and software to generate a product formulation optimized for the following: RBV, raw material cost, optimum fat-to-protein ratio, etc.

The proposed yogurt design procedure using test organisms for creating enriched fermented milk products led to the development of an enriched food product design process with some extra evaluation criteria. The researchers also suggested functional food additives for a new fermented dairy product, the additive amounts, and their effects on the finished product properties. Using biotesting, they estimated the RBV, a comprehensive optimization criterion, for a fermented dairy product with functional food ingredients: lactulose, multivitamin premixes, antioxidants, and polyunsaturated fatty acids. The rheology properties (effective viscosity, moisture holding capacity) of the enriched fermented dairy product were also studied. In addition, a computer application for making enriched fermented dairy product formulations was created [31].

Still, the results applied only to yogurt and fermented dairy beverages (also enriched with functional ingredients) made with other types of lactic acid bacteria. There is a category of products that differs significantly in its physical and chemical properties from those described above.

One such product is curd. Its distinctive features are high protein content, low lactose content, and whey separation after fermentation. The fat content can be adjusted to obtain high-fat or light curd varieties. The key advantage of curd is its protein content. This is due to its amino acid composition. The curd protein contains all the amino acids essential for the human body.

Furthermore, the curd is the most balanced whole-milk product in terms of healthy nutrition; its ratio of proteins, fats, and carbohydrates is 1:1:1.5 [32].

The purpose of this study is the adaptation of the RBV evaluation procedure for the curd/curd product manufacturing processes.

## 2. Materials and Methods

We conducted the research at the Whole-Milk Products Manufacturing Lab, Russian National Diary Research Institute.

We studied curd (cottage cheese) and curd products.

### 2.1. Curd Manufacturing

For all experiments, we used raw milk from Russian Black Pied cattle (mass fraction of fat—3.9%; protein—3.5%, acidity—17 °T). The curd produced was acid curd and fermented curd.

Acid curd production (acid method):

The milk is pasteurized at 78 ± 2 °C for 20 s. After pasteurization, milk is cooled down to 30 ± 2 °C, and the *Lactococcus lactis* subsp*. lactis* starter culture is added (3 wt.% of the milk weight). The fermentation time at 30 ± 2 °C varies from 8 h to 12 h. After fermentation, the curd is sliced and heated for whey separation to 45 ± 2 °C for 30–40 min. Then, the curd is cooled and poured into a self-pressing mold for 1 h to 4 h. The final product is cooled and packed.

Fermented curd production (acid-rennet method):

It is similar to acid curd production except for the following:

- There is no curd heating step for whey separation;
- The starter culture and calcium chloride enzyme rennet (enzyme composition: chymosin 70%, beef pepsin 30%) are also added.

Table 1 lists the required organoleptic properties of the curd.

The expected physical and chemical properties are listed in Table 2.

### 2.2. Assessment of Physical, Chemical, and Rheology Properties

We used the standard procedure to determine the chemical composition of the finished product. The fat content was determined by the Gerber method (ISO 2446:2008). The total protein was estimated by the Kjeldahl method using a KJELTEC system (ISO 8968-1:2014).

The moisture mass fraction in the finished product was estimated by accelerated drying of a product sample (3–5 g) at 150 ± 2 °C.

We used a Petrotech penetrometer (Germany) to measure the rheology properties at a 0.08 kg constant penetration force, 23 ± 0.5 °C, and for 5 s.

The ultimate shear stress was estimated with the Rebinder equation:

(2) $$\Theta_{o}=\frac{K\cdot m}{h_{max}^{2}},\;\mathrm{Pa}$$

where *K* is the cone (penetrator apex angle) constant, N/kg; *m* is the weight of the movable device components, kg; and $h_{max}$ is the max cone penetration depth, m.

In order to compare the results, the experimental and reference samples were taken from the same batch made with the same raw milk. Number of experiments: 3–5. Unless otherwise specified, the statistical significance of the results was assessed with the Student’s *t*-test at 0.95 confidence level.

### 2.3. Express Biotesting of Curd Relative Biological Value

The test organisms were *Tetrahymena pyriformis* (Wh-18 strain). The test procedure was described in our earlier paper [30]. The relative biological value is determined by the ratio of the number of infusoria grown on the test sample to the number of infusoria grown on the control sample, expressed in %. The previously examined, fermented dairy products significantly differed from the curd in physical, chemical, structural, and mechanical properties. For this reason, the RBV evaluation required a modification of the sample preparation procedure. Two options were tested. The first option was the preparation of a suspension containing 1 g of the tested curd and 9 mL of distilled water, subsequently poured into a carbohydrate–salt yeast culture medium (CSY) consisting of 1.5 g of glucose, 0.1 g of yeast extract, 0.1 g of sodium chloride, and up to 100 mL of distilled water. The second option consisted of the CSY and distilled water introduced into a jar with 1 g of the curd test sample. The results indicated that the second option was preferable.

### 2.4. Curd Product Components Selection

Since there is a demand not only for curd but for a variety of curd products (paste, soufflé, whipped products, etc.), we also studied the selection of components (curd + fermented milk beverage) to make a curd product enriched with functional ingredients. For this purpose, we used a fermented dairy beverage; non-fat milk was pasteurized at a temperature of 92 ± 2 °C with an exposure time of 10 min. Further, the mixture was cooled to fermentation temperature, and it was fermented. For the reliability of the results, we used the same *Lactococcus lactis* subsp*. lactis* starter culture, 5 wt.% of the milk weight. The non-fat milk was fermented at 30 ± 2 °C for 8 h to an acidity of ≥75 °T (pH 4.5–4.6). The fermented milk clot obtained was partially cooled and mixed. Further, the products were packed in consumer packaging, hermetically sealed, and sent for post-cooling structure formatting for 16 h and were stored in a refrigerator at a temperature of 4 ± 2 °C.

### 2.5. Statistical Analysis

All experiments were performed in triplicate at least. The tables report means and standard deviation. Unless otherwise specified, the experiment data were subjected to ANOVA with post hoc Tukey test to discover significantly different means. Significance level was set to 0.05.

## 3. Results

**Figure 1:**
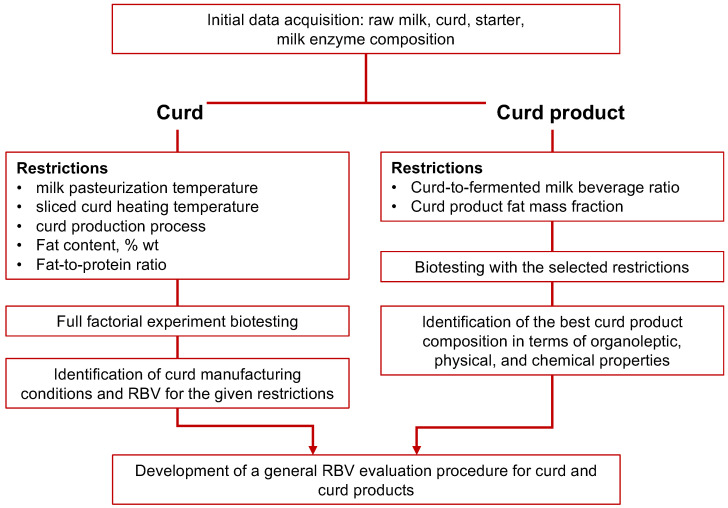
Modification of biotesting-based, fermented dairy product design for curd and curd products.

Figure 1 shows the process flowchart we used in the research. In the first stage, we created a database of basic raw materials: milk, curd, starters, milk enzymes, chemical composition, and functional properties.

### 3.1. Curd

In the next step, we determined: the milk pasteurization temperature ranges; sliced curd heating temperature; fat mass fraction; and fat-to-protein mass fraction ratio. We produced two types of curd: acid and fermented.

In order to estimate the effects of the milk pasteurization temperature on the curd properties, we studied the composition of acid curd made from milk pasteurized at 78 ± 2 °C (recommended by the curd production guidelines) and 88 ± 2 °C (elevated to increase the yield) (Table 3).

The table shows that the criterion sought (RBV) in the curd made from pasteurized milk at 88 ± 2 °C decreased by 33.6%.

**Figure 2:**
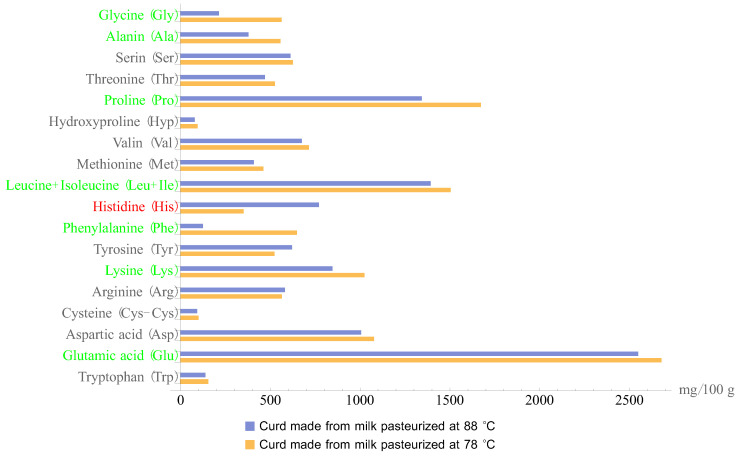
Amino acid composition of the acid curd samples made from the milk pasteurized at 78 ± 2°C and 88 ± 2 °C. Red: amino acids with content that was higher in the samples made from the milk pasteurized at 88 ± 2 °C, green: 78 ± 2 °C. Black: amino acids without statistically significant difference in the content.

The same trend was observed when examining the amino acid composition of the curd made from milk pasteurized under the specified conditions (Figure 2).

For further studies, the pasteurization temperature was set to 78 ± 2 °C.

In order to investigate the effect of the curd heating conditions on the acid curd properties, the following temperatures were selected: 45 ± 2 °C, 50 ± 2 °C, 55 ± 2 °C, and 60 ± 2 °C.

**Figure 3:**
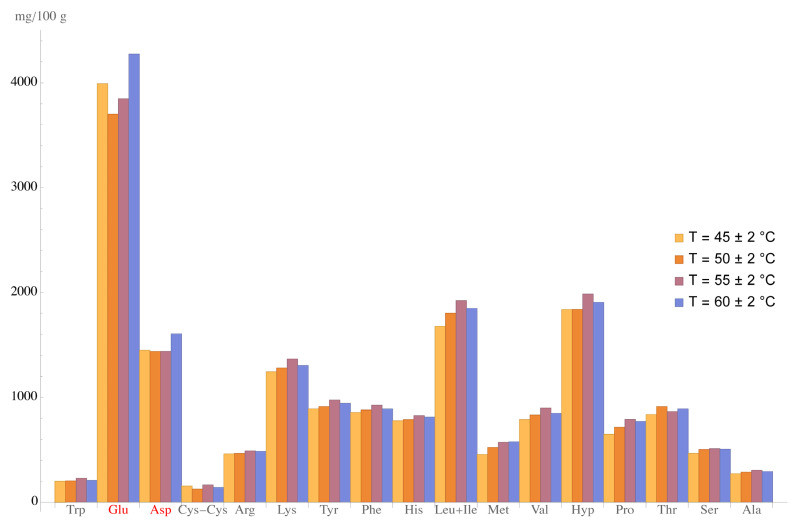
Amino acid profiles of the acid curd samples, heating temperatures: 45 ± 2 °C, 50 ± 2 °C, 55 ± 2 °C, 60 ± 2 °C. Red: amino acids with content in the sample made at 60 ± 2 °C that was statistically different from the other samples (ANOVA test and Tukey’s post hoc test).

Table 4 and Figure 3 show the physical, chemical, and biological properties of the acid curd samples and their amino acid composition.

Their number of amino acids tended to increase as the curd was heated up to 60 ± 2 °C.

Next, we studied the differences between acid and fermented curd.

**Figure 4:**
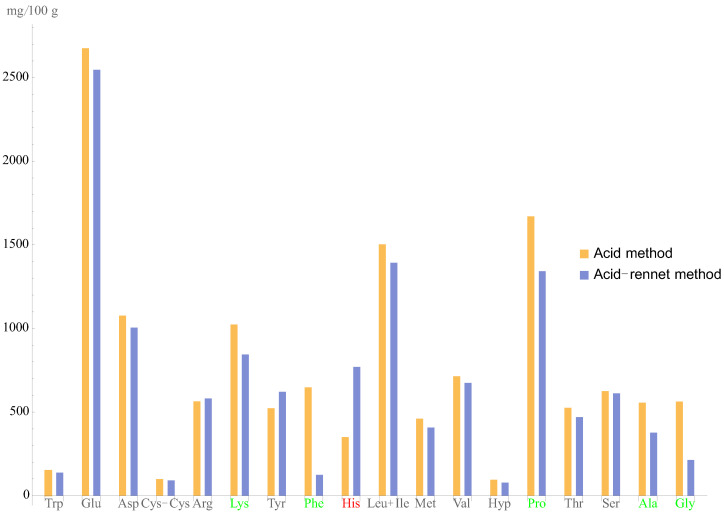
Amino acid profiles of the acid and fermented curd samples. Red: amino acids with content that was higher in the fermented curd samples. Green: amino acids with content that was higher in the acid curd samples. Black: no significant statistical difference in amino acid content.

Table 5 and Figure 4 show the physical, chemical, rheology, and biological properties and the amino acid composition of the acid and fermented curds.

Since the fat mass fraction was also a limitation, the next step was to determine the effects of fat content on the physical, chemical properties, and bioavailability of the curd samples (Table 6).

The curd sample properties examined were within the rated ranges. The greatest RBV increase was found in the 18 wt.% fat curd.

For the purposes of biotesting, we suggest measuring the fat mass fraction and the relationship between the fat-to-protein ratio and RBV. The fat-to-protein ratio in non-fat curd is 0.026 (which corresponds to the 0.5% max acceptable fat content); for 5% fat curd: 0.31; 9% fat curd: 0.58; and 18% fat curd: 1.26. The RBV values (Table 6) indicate that the optimum fat-to-protein mass fraction ratio is 1.26.

With the experiments, we identified the two most significant constraints for the subsequent two-factor experiment: milk pasteurization temperature and curd heating temperature after fermentation. These restrictions apply to acid curd. In order to study the relationship between biological value and the curd manufacturing variables, we developed a full factorial quadratic experiment design (Table 7). The input parameters are the milk pasteurization temperature and the curd heating temperature.

**Figure 5:**
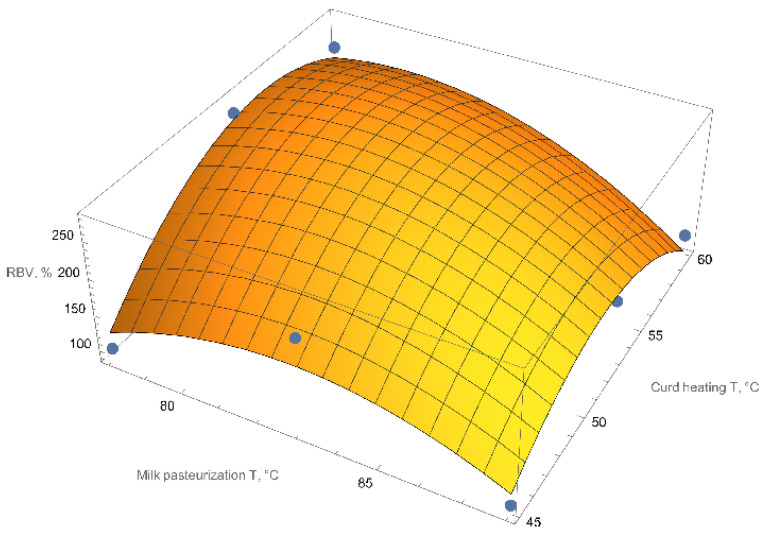
The RBV response surface as a function of the milk pasteurization temperature (°C) and curd heating temperature (°C). Blue dots: the full factorial experiment results.

In order to verify that the accuracy of the experiments was identical, we estimated the variance of reproducibility and the Cochran’s C test. The resulting equation was verified with Fisher’s criterion. The experimental data confidence probability was set to 0.05. The significance of the regression equation coefficients was tested by Student’s *t*-test. All the regression coefficients were significant at *p* < 0.05. The final expanded equation was:

(3) $$\begin{array}{l} RBV\left(T_{mp},T_{ch}\right)=\\ =-22648.9+418.81\;T_{mp}-2.3\;T_{mp}^{2}+220.25\;T_{ch}-0.83\;T_{mp}T_{ch}-1.42\;T_{ch}^{2}, \end{array}$$

where *RBV* is the resulting biological value, $T_{mp}$ is the milk pasteurization temperature, and $T_{ch}$ is the curd heating temperature. Figure 5 shows this relationship as a response surface.

As a result of the full factorial experiment, we identified the optimal curd production conditions to maximize the RBV: 81 °C milk pasteurization temperature and 54 °C curd heating temperature for acid curd. The theoretically achievable biological value under these conditions is 282%.

### 3.2. Curd Product

We decided to limit the range of components used in curd products. Table 8 shows the curd product components and the RBV values.

**Figure 6:**
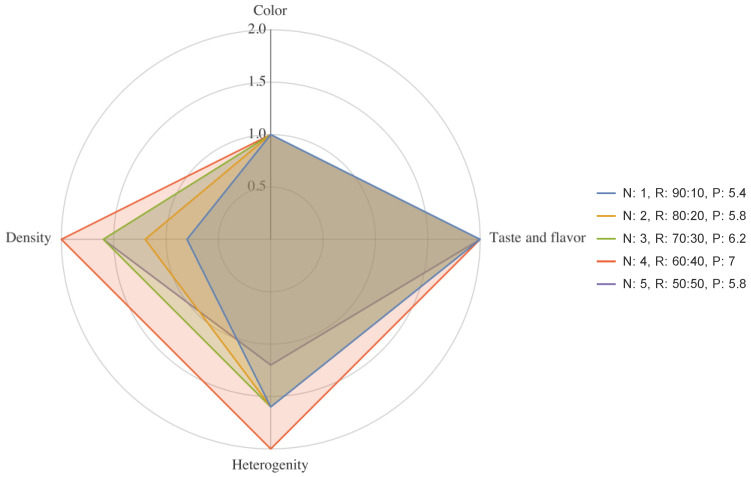
Diagram of the curd product organoleptic properties. N: sample group number, R: curd-to-fermented-dairy-beverage ratio, *p*: final score, points.

Next, we assessed the organoleptic properties of the new curd product (Table 9). We used a five-point scale to estimate the organoleptic properties of the samples (Figure 6).

Sample No. 4 (non-fat acid curd: 60%, fermented dairy beverage: 40%) had the highest organoleptic score and the highest RBV. The advantages of the sample were its high homogeneity, moderately spreadable texture, and that it had no solid particles.

Then, we divided the curd product samples into four groups by their fat content: 0.5 wt.%, 5 wt.%, 9 wt.%, 18 wt.%, and non-fat fermented dairy beverage (0.05% fat, 3% protein). The ratio of the samples was 60 to 40. Table 10 lists the physical, chemical, and biological properties of the samples. Sample No. 1, made from non-fat curd and fermented dairy beverage, served as a reference.

Table 11 lists the structural and mechanical properties (moisture holding capacity and ultimate shear stress).

## 4. Discussion

We refined the curd/curd product biotesting procedures to identify the effects of the curd composition and production methods on the relative biological value (RBV). As experiments, we applied physical, chemical, biotechnological, and rheology analyses.

The first restriction analyzed was the effect of milk pasteurization conditions on curd properties.

The 78 ± 2 °C optimal milk pasteurization temperature has been confirmed by several Russian National Diary Research Institute studies. Pasteurization is a critical curd production stage affecting the quality and safety of the final product. The condition of the proteins and the whey separation process are also important. Increasing the pasteurization temperature above 80 °C results in dense curd formation during fermentation due to increased casein hydration and slower whey separation.

As the pasteurization temperature is reduced to 71 ± 2 °C, the serum proteins are not sufficiently coagulated, resulting in a reduced total protein fraction as the serum is separated. The final product yield is also decreased. Additionally, at low pasteurization temperatures, there is a higher risk of fat rancidity during storage. At low pasteurization temperatures, cases of incomplete inactivation of thermostable milk enzymes are possible. Enzymes which have retained their activity can cause undesirable biochemical processes in milk and dairy products, thus, reducing the quality and biological value of milk. In particular, lipase can cause rancidity of dairy products [33].

Despite the above flaws, in order to increase the final product yield, manufacturers increase the milk pasteurization temperature to 88 ± 2 °C. We studied both the optimal 78 ± 2 °C and elevated 88 ± 2 °C milk pasteurization temperatures.

The decrease in basic amino acids in the curd made from the milk pasteurized at 88 ± 2 °C can be explained by the fact that, in this pasteurization condition, free amino acids are formed. The amount of non-protein nitrogen is increased as the free amino acids are converted into it or the amino acids react with each other.

Setting the right sliced curd heating temperature is important for the acid curd production as it accelerates the whey separation.

Since syneresis is affected by many factors acting not only during the curd heating but also during the self-pressing, Vlodavets et al. [34] obtained the following theoretical relationship to describe the kinetics of syneresis completely:

(4) $$\frac{dV_{t}}{dt}=\frac{K}{\sqrt{t}}\;\left(V_{k}-V_{t}\right),$$

where *t* is the duration of syneresis; $V_{t}$ is the whey volume during the period of syneresis; $V_{k}$ is the max volume of whey at the end of syneresis; and *K* is the factor describing the physical and chemical properties of the curd.

Since it is challenging to estimate *K* with sufficient accuracy, the amount of whey separated during pressing and self-pressing is experimentally determined ad hoc.

The optimal curd temperature before slicing is 40–45 °C. Just as in the case of milk pasteurization, many manufacturers raise the curd heating temperature to 60 °C to intensify the process.

In order to select the optimal heating conditions, we should study their effects on the amino acid composition, the amount of non-protein nitrogen, serum proteins, and remaining proteins in the serum.

The above-mentioned research investigated the effect of acid and fermented curd production processes on the RBV values. The key difference, apart from the components used, was the protein coagulation.

For acid curd, lactic acid bacteria process lactose to make lactic acid. Acidic coagulation of proteins occurs in this way. To intensify the whey separation process, the curd should be heated after slicing [35].

For fermented curd, milk enzymes and calcium chloride are introduced, resulting in protein coagulation. Therefore, no curd heating is required.

Then, we studied the effect of the fat mass fraction on the RBV value. For the curd, this restriction was considered both individually and as the fat-to-protein mass fraction ratio. The reason was that, according to studies, the fat-to-protein mass fraction ratio affects digestibility [36]. A low-fat diet results in the following: consumed proteins are not deposited in the body but are used as an energy source; and protein digestibility is reduced with the subsequent formation of protein metabolic products (uric acid, etc.). Therefore, in this case, it is better to estimate the fat-to-protein ratio in the curd. The study results (Table 6) confirm that the required curd fat-to-protein ratio should be maintained.

A curd product enriched with functional ingredients should be microbiologically safe. For safety purposes, the ingredients are added to the normalized mixture before pasteurization. During fermentation and whey separation, some of the functional additive soluble components enter the whey. Therefore, the most acceptable option for curd enrichment is designing a curd product containing curd and a fermented dairy beverage enriched with functional ingredients.

With the organoleptic evaluation and biotesting of the samples, we identified the ratio of fermented dairy beverage (40%) and acid curd (60%) with RBV = 142 ± 9.1%. This ratio can be used in new curd products with various fat content.

It should be noted that the biotesting of the curd product samples produced ambiguous results. Sample group No. 2 (Table 10) made from the 5 wt.% fat curd and a non-fat, fermented milk beverage showed an RBV increase, while the samples made from the 18 wt.% curd had a statistically significant decrease in RBV. As we measured the curd RBV (refer to Table 6), there was a *Tetrachymena* increase in fat 5 wt.%, 9 wt.%, and 18 wt.% curd samples.

## 5. Conclusions

Using the express method for assessing the RBV and the selected optimality criteria, in this study, we adapted the methodology for the RBV assessment of fermented milk products developed by the authors for the technologies of curd and curd products. The parameters of the technological process for the production of curd, which were chosen as limitations, were analyzed: milk pasteurization temperature; clot heating temperature after cutting; ways of producing curd; the ratio of the mass fraction of protein and fat in curd; and the mass fraction of fat in curd. Based on the experiments performed, the most significant parameters are the milk pasteurization temperature and the clot heating temperature. As a result of a full factorial experiment, the optimal regime for obtaining curd for maximizing the RBV was established. The temperature of pasteurization of milk is 81 °C, and the temperature of heating the curd is 54 °C with the acid method of producing curd. This regime, all other things being equal, provides an RBV of at least 282%. In addition, the biotesting method confirmed the percentage of components for the design of curd products. A methodology for designing effective technologies for curd and curd products was developed. It is quite possible that the results obtained would differ if the research is continued with more raw milk or if other types of milk (for example, reconstituted) or other types of curd obtained by separation or ultrafiltration or heat-treated curd products (with a long shelf life) are investigated. Despite this, the results obtained can be used in the production of curd currently produced by enterprises on existing equipment.

## Figures and Tables

**Table 1:** Organoleptic properties of the curd (ISO 31453-2013).

Property	Value
Appearance and texture	Soft, slightly spreadable, or crumbly texture, with or without perceptible milk protein particles.
Taste and flavor	Pure, fermented milk, no other flavors or odors.
Color	White and creamy, consistent.

**Table 2:** Physical and chemical properties of the curd (ISO 31453-2013).

	Types of Curd by Fat Content			
**Fat Content, %, Not Less**	**Fat-Free**	**5.0**	**9.0**	**18.0**
Moisture mass fraction, %, max	80.0	75.0	73.0	65.0
Protein mass fraction, %,not less	18.0	16.0	16.0	14.0
Acidity, °T, not more	240	230	220	210
Phosphatase or peroxidase	Absent			

**Table 3:** Physical, chemical, and biological properties of acid curd samples. Protein and RBV mass fractions at different temperatures showed a statistically significant difference (marked with different superscript letters).

Skim Milk Pasteurization Temperature, °C	Protein, wt. %	Moisture, wt.%	RBV, %
78.0 ± 2.0	17.12 ± 0.28 ^a^	78.5 ± 5.5	100.0 ^a^
88.0 ± 2.0	16.58 ± 0.21 ^b^	79.5 ± 5.8	66.4 ± 4.6 ^b^

**Table 4:** Physical, chemical, and biological properties of the acid fat-free curd samples. ^a–c^: Means in columns which do not share the same superscript letter were significantly different.

Curd Heating Temp. of the Fat-Free Curd	Protein, wt.%	Moisture, wt.%	RBV, %
45 ± 2 °C	16.87 ± 0.17 ^a^	79.5 ± 3.5	100.0 ^a^
50 ± 2 °C	17.43 ± 0.21 ^b^	78.5 ± 4.7	80.5 ± 5.6 ^b^
55 ± 2 °C	18.15 ± 0.33 ^c^	78.1 ± 5.3	45 ± 3.15 ^c^
60 ± 2 °C	18.23 ± 0.27 ^c^	78.0 ± 5.6	44.1 ± 3.1 ^c^

**Table 5:** Physical, chemical, rheology, and biological properties of the acid and fermented curd samples. ^a,b^: Means in columns which do not share the same superscript letter are significantly different.

Sample	Protein, wt.%	Moisture, wt. %	Penetration Depth, m	Ultimate Shear Stress *Θₒ*, Pa	RBV, %
Fat-free (acid curd)	18.11 ± 0.9	72.0 ± 3.7	0.034 ± 0.002	4147.06 ± 207.35	100.0 ^a^
Fat-free (fermented curd)	17.47 ± 0.8	72.7 ± 5.3	0.035 ± 0.002	4028.06 ± 201.4	64.7 ^b^

**Table 6:** Properties of curd samples with different fat content. ^a–c^: Different superscript letters mark significant statistical difference between means of RBV.

Sample	Protein, wt.%	Moisture, wt.%	RBV, %
Non-fat (0.5 wt.% fat) (Reference) (acid curd)	18.89 ± 1.32	72.5 ± 5.07	100.0 ^a^
5 wt.% fat curd (acid curd)	16.07 ± 1.12	72.0 ± 5.04	125.0 ± 8.75 ^b^
9 wt.% fat curd (acid curd)	15.47 ± 1.01	70.0 ± 4.9	126.0 ± 8.82 ^b^
18 wt.% fat curd (fermented curd)	14.26 ± 0.98	56.0 ± 3.94	150.0 ± 10.5 ^c^

**Table 7:** Full factorial quadratic experiment design.

Test No.	Milk Pasteurization T, °C	Curd Heating T, °C	RBV, %
1	88 ± 2	60 ± 2	111 ± 5.5
2	88 ± 2	45 ± 2	95.5 ± 4.7
3	78 ± 2	60 ± 2	239 ± 11.9
4	78 ± 2	45 ± 2	100 ± 7.5
5	83 ± 2	60 ± 2	175 ± 8.75
6	83 ± 2	45 ± 2	213 ± 10.65
7	88 ± 2	53 ± 2	170 ± 8.5
8	78 ± 2	53 ± 2	264 ± 13.2

**Table 8:** Curd product samples’ components. ^a,b^: Different letters mark sample groups with statistically distinguishable RBV mean values.

Sample No.	Fat-Free Acid Curd	Fermented Dairy Beverage	RBV, %
No. 1	90	10	100 ± 3.35 ^a^
No. 2	80	20	105 ± 5.35 ^a^
No. 3	70	30	91 ± 4.45 ^a^
No. 4	60	40	142 ± 9.1 ^b^
No. 5	50	50	131 ± 7.55 ^b^

**Table 9:** Organoleptic properties of the curd product.

Property			Points to Subtract (from Max Score of 5)
1. Color (weight: 0.2)	Specific, milky-white or cream-colored, no defects		0
	Non-specific tone	*not pronounced*	1
		*weak*	2
		*clear*	3
		*pronounced*	4
	Non-specific color		5
2. Taste, flavor (weight: 0.4)	Fermented milk taste and flavor, expressed, pure		0
	Fermented milk, not pronounced, flat taste		1
	Excessively acid taste and flavor		2
	Off-flavor	*weak*	2
		*clear*	3
		*pronounced*	4
	Non-specific taste		5
3. Texture, appearance (weight: 0.4)	Homogeneous, without defects		0
	Homogeneity deviations	*slightly non-homogeneous*	1
		*lumpy, friable, insignificant whey separation*	2
		*non-homogeneous, crumbly*	3
		*non-homogeneous, pronounced protein particles and syneresis*	4
	Significantly non-homogeneous		5
	Paste-like, spreadable, soft, moderately dense		0
	Density deviations	*soft, insufficient or excessive density, too viscous*	1
		*insufficient or excessive density*	2
		*liquid or very viscous, very dense*	3
		*liquid, stratified, coarse*	4
	Mushy		5

**Table 10:** Physical and chemical properties of curd product samples. ^a–c^: Different letters mark statistically significant RBV mean differences.

Group Number	Fat wt.%	Protein, wt.%	Moisture, wt.%	RBV, %
1	0.08	12.0 ± 0.8	84.42 ± 5.9	100.0 ± 5.6 ^a^
2	3.0 ± 0.2	10.8 ± 0.7	82.72 ± 5.8	121.8 ± 7.51 ^b^
3	5.4 ± 0.4	10.6 ± 0.7	80.32 ± 5.6	94.5 ± 4.32 ^a^
4	10.8 ± 0.7	9.6 ± 0.6	75.64 ± 5.3	63.6 ± 2.52 ^c^

**Table 11:** Rheology, structural, and mechanical properties of curd products.

Group Number	Moisture Holding Capacity, %	Relative Penetration, m	Ultimate Shear Stress *Θₒ*, Pa	Sample No.
1	86.88 ± 6.08	0.0180	573.17 ± 40.12	1
2	80.8 ± 5.65	0.0172	507.19 ± 35.49	2
3	79.1 ± 5.53	0.0167	476.35 ± 32.71	3
4	74.0 ± 5.18	0.0157	435.18 ± 30.46	4

## Data Availability

Data is contained within the article.
